# Independent and Joint Associations of Pessimism, Total Calorie Intake and Acid-Producing Diets with Insomnia Symptoms among Breast Cancer Survivors

**DOI:** 10.3390/jcm11102828

**Published:** 2022-05-17

**Authors:** Tianying Wu, Cesar Arevalo, Fang-Chi Hsu, Suzi Hong, Humberto Parada, Mingan Yang, John P. Pierce

**Affiliations:** 1 Division of Epidemiology and Biostatistics, School of Public Health, San Diego State University, San Diego, CA 92182, USA; carevalo1352@sdsu.edu (C.A.); hparada@sdsu.edu (H.P.J.); myang@sdsu.edu (M.Y.); 2 Moores Cancer Center, University of California, San Diego, CA 92037, USA; jppierce@health.ucsd.edu; 3 Department of Biostatistics and Data Science, Wake Forest School of Medicine, Winston-Salem, NC 27101, USA; fhsu@wakehealth.edu; 4 Department of Psychiatry, School of Medicine, University of California, San Diego, CA 92093, USA; s1hong@health.ucsd.edu; 5 Herbert Wertheim School of Public Health and Human Longevity Science, University of California, San Diego, CA 92093, USA

**Keywords:** pessimism, dietary, sleeping disorders, breast cancer survivors

## Abstract

Insomnia is prevalent in up to 40% of breast cancer survivors. Few studies have examined pessimism and dietary factors as risk factors for insomnia among breast cancer survivors. We leveraged a cohort of 2944 breast cancer survivors who enrolled in the Women’s Healthy Eating and Living study; these survivors provided dietary, insomnia, mental health, demographic, and lifestyle information at baseline and at 1- and 4-year follow-up assessments. Insomnia symptoms were assessed using the Women’s Health Initiative (WHI)-Insomnia Rating Scale, and pessimism was assessed using the Life Orientation Test Revised (LOT-R). Total calorie intake and acid-producing diets were assessed using 24 h dietary recalls. Multivariable-adjusted generalized estimating equation (GEE) models were used to test the independent and joint effects of psychological and dietary factors on insomnia. In the multivariable model, women in the third tertile of pessimism had greater odds (OR = 1.57 95% CI [1.37–1.79]) of insomnia when compared to women in the lowest tertile. Total calorie intake and acid-producing diets were each independently and significantly associated with insomnia symptoms. Further, pessimism and calorie intake/acid-producing diets were jointly associated with insomnia. For instance, women with pessimism scores in tertile 3 and total calorie intakes < median reported 2 times the odds (OR = 2.09; 95% CI [1.51–3.47]) of insomnia compared to women with pessimism score in tertile 1 and calorie intakes < median. Our results highlight the need for patient care regarding mental health, and recommendations of healthy dietary intakes for breast cancer survivors.

## 1. Introduction

According to the World Health Organization (WHO), breast cancer is one of the leading cancers impacting individuals worldwide, with 2.26 million new cases diagnosed in 2020 [1]. In the United States, breast cancer accounts for approximately 30% of all new female cancers diagnosed each year [2]. Insomnia is highly prevalent among individuals with cancer [3]. Data from the National Health Interview Survey showed that 30% of cancer survivors present with insomnia, significantly higher than those without a history of cancer [4]. The prevalence of insomnia and sleep disorders among breast cancer survivors is much higher (up to 40%) than among other cancer survivors [5]. Hence, it is important to identify risk factors that may influence insomnia to help improve the quality of life of breast cancer survivors.

A number of studies have examined risk factors of insomnia among women with breast cancer. For example, chemotherapy, aging, and menopause have been linked to sleep disorders [6,7,8]. Furthermore, emotional disturbance is a significant contributing factor to insomnia symptoms [9,10,11,12]. For instance, depression contributes to worsening insomnia symptoms, whereas optimism may have a protective effect on sleep disorders [12,13]. Pessimism, an attitude opposite to optimism, is defined by the American Psychological Association (APA) as “the attitude that things will go wrong and that people’s wishes or aims are unlikely to be fulfilled” [14]. Pessimism is a cognitive attribute of depressivity and is consistently associated with depression [15]. The depression–insomnia link has been widely studied [9,16]; however, the association between pessimism and insomnia symptoms or sleep health has not drawn much attention nor been well-studied among cancer survivors.

Among other risk factors for insomnia, diets may also have important impacts on sleeping disorders. Foods high in glycemic index, protein, carbohydrate, trans fatty acids and sodium, and low in vegetables may increase the likelihood of insomnia [17,18,19]. Additionally, increased total calorie intake was associated with an increased risk of insomnia in men [19], but this association has not been examined in women with breast cancer. Diets with high intakes of protein (e.g., meat) and low intakes of fruit and vegetables are acid-producing diets. Our group has shown that breast cancer survivors are susceptible to acid-producing diets, resulting in an increased risk of mortality, depression, and reduced physical function [20,21,22,23]. Cancer survivors have a lower capacity to adjust acid-base balance than the general population [24,25]. However, the association between acid-producing diets and insomnia in breast cancer survivors has not been assessed.

In this study, we leveraged resources from a randomized trial, —the Women’s Healthy Eating and Living (WHEL) study, which collected dietary and psychological factors and insomnia information among breast cancer survivors at baseline and during follow-up. Our primary aim was to determine the independent and joint impact of pessimism, total calorie intake, and acid-producing diets on insomnia among breast cancer survivors.

## 2. Materials and Methods

### 2.1. Study Design

The WHEL has been previously described [26]. In brief, recruitment occurred from 1995 to 2000, and participants of the study were recruited from various clinical centers in Oregon, California, Arizona, and Texas. The study recruited 3088 breast cancer survivors who were diagnosed with stage I, II, or IIIA at diagnosis and who were diagnosed within the previous four years. The purpose of the original study was to conduct a randomized trial to compare cancer recurrence and total mortality between the intervention group and the control group. The intervention group followed a diet high in fruit, vegetables, and fiber and low in total fat (no more than 20% of fat from total energy), and the control group followed a general recommended diet by the U.S. Department of Agriculture and the National Cancer Institute at the time of intervention. We analyzed the study’s data as a cohort study controlling the intervention status, given that the original trial did not significantly improve breast cancer prognosis. The cohort was followed for six years. Data on dietary information, mental health, and physical health, including insomnia symptoms or cancer recurrence, were collected at baseline, and at follow-up assessments, which occurred after 1 and 4 years after enrollment.

This study was approved by the Institutional Review Board (IRB) at the University of California at San Diego and other participating centers. Since we used de-identified data provided by the principal investigator of the WHEL study, our study was exempt from IRB review at San Diego State University (protocol number: Temp-1286).

### 2.2. Assessment of Insomnia Symptoms

The WHEL study measured insomnia symptoms at baseline and each follow-up using the five-item Women’s Health Initiative-Insomnia Rating Scale (WHI-IRS), a validated measure with a high internal consistency [27,28]. The five items included questions related to having difficulty initiating sleep, waking several times throughout the night, early waking, trouble resuming sleep, and the overall sleep quality in the past four weeks. A score greater than nine on the WHI-IRS can classify an individual as having clinically significant insomnia.

### 2.3. Assessment of Pessimism

The Life Orientation Test Revised (LOT-R) measures both pessimism and optimism and consists of six questions. It can measure optimism and pessimism on the same scales, with higher scores indicating a more optimistic personality [29]. Pessimism was treated as an independent construct and was assessed based on the three questions related to pessimism. The questionnaire included the following items “If something can go wrong for me, it will”, “I hardly ever expect things to go my way”, and “I rarely count on good things happening to me” on a five-point Likert scale from strongly disagree to strongly agree. Points for these three questions were added to calculate the pessimism score, ranging from 3 to 15. Pessimism was treated independently from overall optimistic trait because the relationship between optimism and pessimism has been shown to differ in the context of physical and psychological health [30].

### 2.4. Assessment of Dietary Intake

Dietary intake was assessed through prescheduled 24 h recalls four times in a three-week time frame. Half of the recalls were conducted on a weekday, while the other half were collected on the weekend. These recalls were conducted at baseline and each follow-up. The multi-pass Minnesota Nutrition Data System software was used for calculating the amount of each nutrient and foods, such as vegetables (NDSR, 1994–2006, University of Minnesota, Minneapolis, MN, USA).

The net endogenous acid production (NEAP) score and the potential renal acid load (PRAL) score were used to measure each participant’s consumption of acid-producing diets. NEAP, developed by Frassetto et al., measures protein and potassium intake [31]. PRAL, in addition to protein and potassium intake, accounts for calcium, magnesium, and phosphorus [32]. PRAL and NEAP scores are used to evaluate the acid-producing potential of a participant’s diet. Higher NEAP and positive PRAL scores indicate a diet with greater acid-producing potential. The following formulas were used to calculate PRAL and NEAP [32].

NEAP (mEq/d) = (54.5 × protein [g/d]/potassium [mEq/d]) − 10.2

PRAL (mEq/d) = (0.49 × protein [g/d]) + (0.037 × phosphorus [mg/d]) − (0.021 × potassium [mg/d]) − (0.026 × magnesium [mg/d]) − (0.013 × calcium [mg/d])

### 2.5. Assessment of Demographic Information

Demographic variables such as age, body mass index (BMI), and smoking status, were collected using self-reported questionnaires. Health conditions, such as diabetes and cardiovascular diseases, were also collected via self-reported questionnaires. Information about breast cancer treatment, tumor hormone receptor status, and patient diagnosis was obtained from medical records. Information used to calculate BMI and any co-morbidities were measured at baseline and at each follow-up.

### 2.6. Statistical Analysis

Statistical analyses were conducted using SAS version 9.4 (SAS Institute Inc., Cary, NC, USA). Differences in baseline characteristics across insomnia status were assessed using a *t*-test for continuous variables or a chi-squared test for categorical variables. The univariate associations of baseline covariates with pessimism score, total calorie intake or dietary acid load score were assessed using chi-squared test for categorical variables or ANOVA for continuous variables.

A Generalized Estimating Equations (GEE) model with binomial distribution, logit link, and exchangeable correlation covariance matrix was used to model the association between total calorie consumption, dietary acid load, and pessimism, and their effect on insomnia. Insomnia was treated as a binary variable (insomnia score ≥ 9 was coded as 1 and score < 9 was coded as 0). The main exposure of interests included pessimism, calorie intake, and PRAL and NEAP scores. For the main analyses, pessimism items were categorized into tertiles and calorie intake and dietary acid load scores were categorized into quartiles. The cut-off points for setting up tertiles or quartiles were based on the average values of three visits (baseline, year 1 and 4). In our multivariable models, we adjusted for the following covariates: caffeine intake, BMI, age, smoking status, status of estrogen and progesterone receptors, co-morbidities, menopausal status, and education.

Multivariable-adjusted GEE models were used to assess the joint impacts of pessimism with total calorie intake/dietary acid load on insomnia scores. The joint impact was assessed by categorizing participants based on their pessimism scores (tertiles) and calorie intake into two categories, using median as the cut-off point, resulting in six groups with a joint classification of pessimism and calorie intake. Similarly, we created six groups based on the joint classification of pessimism and PRAL.

## 3. Results

### 3.1. Baseline Characteristics by WHI-IRS

Table 1 shows the baseline characteristics comparing participants without clinical insomnia (insomnia score < 9) and those with clinical insomnia (insomnia score ≥ 9). Participants with higher inter-quartile ranges of pessimism score and higher median scores of PRAL and NEAP were more likely to present with clinical insomnia score; *p*-value was < 0.001 for pessimism, 0.02 for PRAL and 0.06 for NEAP. Participants in the intervention group and those who had some college education or higher were less likely to present with clinical insomnia (*p*-value ≤ 0.05 for all comparisons). Participants who were post-menopausal (*p*-value ≤ 0.001) and normal weight (*p* value = 0.07) were less likely to present with clinical insomnia at baseline.

### 3.2. Baseline Characteristics by Pessimism 

Table 2 displays the association of baseline characteristics with each tertile of pessimism. Participants in the highest tertile of pessimism were more likely to meet medical definitions of overweight and obese, estrogen receptor (ER)-positive, and progesterone receptor (PR)-positive, be current smokers and alcohol abstainers, and have some college education or higher (*p*-value ≤ 0.05 for all comparisons). Participants in the third tertile of pessimism were more likely to have a higher insomnia score when compared to those in the first tertile (*p* < 0.0001).

### 3.3. Baseline Characteristics by Calorie Consumption

Table 3 displays the association of baseline characteristics with the quartiles of calorie intake. The highest quartile was the participants who were more likely to have a normal weight and be post-menopausal and abstainers of alcohol (*p*-value ≤ 0.05 for all these comparisons). All other associations were not statistically significant.

### 3.4. Baseline Characteristics by PRAL Quartile

Table 4 displays the baseline characteristics with the PRAL quartiles. Participants in the highest quartile of PRAL were more likely to have higher insomnia scores when compared to the lowest quartiles (*p* = 0.002). Participants in the highest quartile of PRAL were more likely to have ER+/PR+, and to have received chemotherapy treatment, be post-menopausal, and have some college or higher education (*p* < 0.001) (*p* < 0.05 for all these comparisons). All other comparisons were not statistically significant.

### 3.5. Longitudinal Impact of Pessimism, Calorie Intake, and Dietary Acid Load

There were 1148 (38.8%), 834 (35.7%), and 722 (35.13%) participants who presented with clinical insomnia at baseline, year 1, and year 4, respectively. As shown in Table 5, in the age-adjusted model, the highest tertile of pessimism had greater odds (OR = 1.64, 95% CI [1.463–1.86]) of presenting with clinical insomnia compared to the lowest tertile. In the multivariable model (Table 5), however, the magnitude of OR was slightly attenuated; the highest tertile of pessimism had greater odds (OR = 1.57 95% CI [1.37–1.79]) when compared to the lowest tertile; we observed a dose–response relationship (*p* for trend < 0.001). Similarly, the odds ratios for total calorie intake, PRAL, and NEAP were not significantly attenuated. In the multivariable model, compared to the first quartile, women in the fourth quartile had 1.14 to 1.24 times the odds of insomnia regarding calorie intake or dietary acid load [OR = 1.14 95% CI (1.00–1.30) for calorie intake; OR = 1.31 95% CI (1.15–1.50) for PRAL, and OR = 1.24 95% CI (1.08–1.42) for NEAP]; *p*-values for trends were 0.02 for both calorie intake and PRAL, but the trend was not significant for NEAP.

### 3.6. Joint Impact of Pessimism and Calorie Intake and Joint Impact of Pessimism and Dietary Acid Load on Presentation of Clinical Insomnia

**Figure 1:**
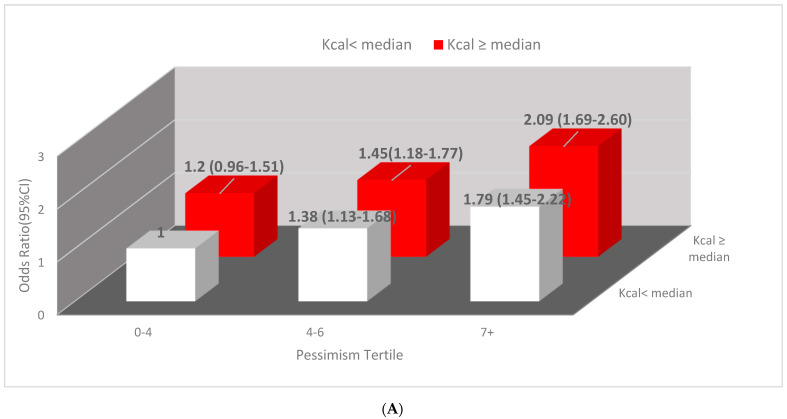

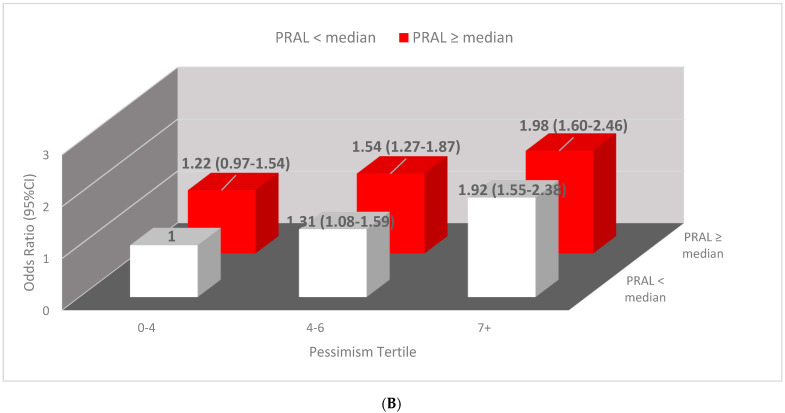
(**A**) **Joint association of calorie intake and pessimism with insomnia.** Covariates in the multivariable-adjusted model include caffeine quartile, BMI, age, smoking status, status of estrogen receptor and progesterone receptor, baseline co-morbidities, menopausal status, and education. (**B**) **Joint association of PRAL and pessimism with insomnia.** Covariates in the multivariable-adjusted model include caffeine quartile, BMI, age, smoking pack and years, estrogen receptor and progesterone receptor, co-morbidities, menopausal status, and education. Abbreviations: PRAL potential renal acid load.

In the multivariable model, we assessed the joint impact of pessimism and calorie intake on insomnia and the joint impacts of pessimism and dietary acid load using PRAL. The results are displayed in Figure 1. As shown in Figure 1A, women within the highest tertile of pessimism and calorie intakes higher than the median (≥1603 Kcal) had two times the odds of insomnia compared to women within the lowest tertile and calorie intake lower than the median (OR = 2.09; 95% CI, 1.68–2.60). Figure 1B displays the joint association of pessimism and PRAL with insomnia. Women whose PRAL was at or above the median and in the highest tertile of pessimism score almost had two times the odds of insomnia, OR = 1.98,95% CI(1.60–2.46), compared to women within the lowest pessimism tertile and PRAL below the median.

## 4. Discussion

The present study investigated both psychological (affective–cognitive) and dietary risk factors associated with insomnia in breast cancer survivors. This longitudinal study demonstrated that a higher pessimism score, increased calorie intake, and dietary acid load scores were independently associated with increased risk of insomnia; however, the magnitude of the association was the largest for pessimism score. Furthermore, our study demonstrated a joint impact of total calorie intake and pessimism on insomnia, and a joint impact of dietary acid load and pessimism on insomnia. Pessimistic individuals with higher calorie intakes or a higher dietary acid load score exhibited almost 2 times the risk of insomnia compared to non-pessimistic individuals with lower calorie intake or a lower dietary acid load score.

Optimism and pessimism are two opposing attitudes and have been found to have different impacts on health outcomes. Optimism has been associated with an overall higher quality of life and sleep quality [12,33]. A sample of women with breast cancer who underwent surgery found that optimism was protective for insomnia [13]. On the other hand, pessimism was associated with lower quality of life scores in breast cancer survivors [34,35]. Though the studies on pessimism and insomnia are scant, depression, to which pessimism is a cognitive attribute, has been found to be strongly associated with insomnia in many studies [36].

The underlying mechanisms for the association between pessimism and insomnia have not been well-studied. Researchers have found that pessimism was associated with insulin resistance [29]; other studies found that pessimism was associated with incidence of coronary heart disease (CHD) [37] and stroke [38]. Insulin resistance [29], or potentially other factors that are associated with CHD (e.g., inflammation), may contribute to pessimism-related sleep disorders. More studies are needed to determine the biological mechanisms behind this.

Prospective studies on acid-producing diet and sleep disorders among breast cancer survivors are limited. Most of the studies on this topic are cross-sectional. Two cross-sectional studies conducted in diabetes and hemodialysis patients found that increased dietary acid load was associated with an increased risk of sleep problems [39,40]. Two other cross-sectional studies among breast cancer survivors found inconsistent results [41,42]. These two studies did not use dietary acid load, which can better assess the quantity of acid-producing diets. In a study conducted in China, high intake of vegetables, which indicate alkaline-producing and low-acid diets, were associated with decreased insomnia symptoms among breast cancer survivors [41]. In contrast, another study showed that individuals with healthy dietary patterns, a diet high in vegetables and low in meat consumption, have greater odds of experiencing insomnia among breast cancer survivors [42]. All these studies are cross-sectional and are subject to reverse causation, which means those with sleeping problems may modify their dietary patterns to a healthy dietary pattern after they experience the onset of sleeping problems. Thus, prospective studies among breast cancer survivors are needed.

Some potential biological mechanisms may help explain why acid-producing diets are associated with worsening insomnia. Lower blood pH can change ventilation and respiratory rhythm, causing repeated sleep disturbances [43] because the acidic environment can change the degree of stimulation of central and peripheral chemoreceptors [43,44]. Breast cancer survivors have a lower capacity to adjust acid–base balance [24,25]; thus, high consumption of acid-producing diets can further exhaust their bodies’ ability to excrete or neutralize acids.

This study is the first to look at the implications of calorie intake on insomnia among breast cancer survivors. Several cohort studies support our finding on the positive relationship between high calorie consumption and insomnia, though they were not conducted in breast cancer survivors. A cohort study among men found that a higher calorie intake was associated with insomnia [19]; however, they did not examine this association in women or women who are breast cancer survivors. Further, the timing of consumption of calorie intake has been studied, where a higher intake of calories in the late evening or night has a negative impact on sleep [45,46] or prognoses among this WHEL cohort [47].

The mechanisms underlying the association between total calorie intake and insomnia are more complex, as they may include bidirectional influences and multi-dimensional risk factors. On the one hand, increased calorie consumption places an extra burden on digestive systems, further reducing the quality of sleep. The reasons are as follows: (1) During sleep, saliva production and swallowing rate reduce, gastric acid secretion significantly decreases, and small intestinal motility diminishes, all of which can significantly lessen the efficiency of food digestion and absorption, especially when someone has a high-calorie meal late at night or has functional dyspepsia, even when having meals at regular hours. (2) Reduced salivary volume and decreased frequency of peristalsis can increase gastric acid reflux. As a result, acid reflux and indigestion can frequently cause sleep arousal [48,49,50,51]. On the other hand, sleep deprivation can lead to an increase in the desire for high-calorie foods [52] because sleep deprivation can change the activities in the brain regions that are related to appetite evaluation and food responses [52]. Sleep disorders may cause some behaviors changes which can further worsen sleep disorders. For instance, short sleep duration creates more time and opportunities to eat and makes people need more energy to sustain extended wakefulness [53,54].

Herein, we report the joint impacts of pessimism and high calorie intake/high acid-producing diets on insomnia, which indicate interactions between pessimism and diets, and that they both can have synergistic impacts on insomnia. In addition, pessimism itself may also influence dietary habits. Individuals with pessimistic personalities are more likely to have an unhealthy diet [55], whereas optimism is associated with a healthy diet [56]. Therefore, the joint impacts of pessimism and diet on insomnia may exceed the addition of each individual effect on insomnia (effect of pessimism on insomnia and effect of the calorie intake/dietary acid load score on insomnia).

This study has several strengths. It is the first longitudinal study to show the independent and joint impact of pessimism and calorie intake/dietary acid load on insomnia. The study enrolled breast cancer survivors from multiple states and had a large sample size with three follow-up periods. Multiple 24 h recalls were collected during each visit, which reduced the within-person variability. While depression and pessimism may affect sleep quality, the rich data set in the WHEL study allowed us to assess pessimism independently from depressive symptoms. Multiple measures of dietary acid load, pessimism, and insomnia scores during follow-up enabled us to conduct time-varying analyses, which are not frequently conducted in other cohorts of breast cancer survivors. The study had some limitations. Participants in the study were primarily white women, limiting the understanding of the relationship across different races. We had repeated measures of diet, pessimism, and insomnia for up to 4 years. However, we were unable to examine the impact of these risk factors beyond four years. We acknowledge that objective measures using polysomnography were not conducted in this study; nevertheless, the insomnia scores have been validated using gold-standard measures of the five-item Women’s Health Initiative-Insomnia Rating Scale (WHI-IRS) [27]. Breast cancer survivors in the WHEL study were recruited during 1995 to 2000 and followed up until 2006. We acknowledge that dietary habits and psychological factors may have been modified in the 2020s; however, the three identified risk factors (pessimism, total calorie intake and acid-producing diets) still exist in today. Therefore, our results need to be confirmed in a contemporary cohort of breast cancer survivors.

## 5. Conclusions

Although depression and its impact on insomnia and other health outcomes have been widely studied, pessimism, a key attribute of depression, has been overlooked in breast cancer survivors. Insomnia is highly prevalent in breast cancer survivors compared to the general population. Therefore, it is essential to improve awareness of pessimism and its independent and joint impact with diet on insomnia among professionals involved in cancer care. Future studies are warranted to replicate these associations in other populations and with other objective measures of insomnia.

## Figures and Tables

**Table 1:** Baseline characteristics of breast cancer survivors by insomnia scores (N = 2960).

	Insomnia Score < 9	Insomnia Score ≥ 9	
	*n* = 1812	*n* = 1148	*p*-Value
Pessimism ^a^	6.0 (4.0, 7.0)	6.0 (5.0, 8.0)	<0.0001
KCAL ^a^	1685.0 (1431.0, 1979.5)	1703.5 (1450.0, 1988.5)	0.30
PRAL ^a^	−4.2(−13.9, 4.4)	−3.0 (−13.4, 5.5)	0.02
NEAP ^a^	39.6 (32.3, 48.3)	40.6 (32.8, 48.8)	0.06
Age at Diagnosis (years) ^a^	50 (45, 57)	50 (45, 57)	0.48
Normal Weight, N (%)	801 (62.9)	472 (37.1)	0.07
White, N (%)	1545 (61.1)	983 (38.9)	0.79
Stage at Diagnosis			
I, N (%)	698 (69.0)	447 (39.0)	0.80
II, N (%)	1019 (61.2)	647 (38.8)	
III, N (%)	95 (63.8)	54 (36.2)	
Intervention Group, N (%)	929 (63.0)	546 (37.0)	0.05
Hormone Receptor Status			
ER+/PR+, N (%)	1109 (60.3)	729 (39.7)	0.35
Post-Menopause, N (%)	1398 (59.4)	954 (40.6)	<0.0001
Chemotherapy, N (%)	1266 (61.4)	797 (38.6)	0.92
Radiation, N (%)	1112 (61.2)	704(38.8)	0.85
Smoking Status			
Never N, (%)	988 (62.0)	606 (38.0)	0.48
Past N, (%)	739 (60.0)	493 (40.0)	
Current N, (%)	85 (63.4)	49 (36.6)	
Education			
Some College or above N, (%)	1015 (63.5)	584 (36.5)	0.006
Alcohol Abstainer N, (%)	571 (61.4)	359 (38.6)	0.97

^a^ Continuous variables are presented as median (inter-quartile range). To test statistical significance between women with insomnia symptoms vs. those without insomnia symptoms, we used a *t*-test for a continuous variable (variables labeled with ^a^, such as pessimism) and chi-squared test for a categorical variable (variables not labeled with ^a^, such as education). Abbreviations: KCAL: Kilo Calorie, ER.: estrogen receptor-positive, PR: progesterone receptor-positive; PRAL potential renal acid load, NEAP net endogenous acid production.

**Table 2:** Baseline characteristics according to pessimism score in the WHEL study (*n* = 2960).

	Pessimism Score			
	Tertile 1	Tertile 2	Tertile 3	*p*-Value
	0–4	5–6	7+	
	*n* = 813	*n* = 1105	*n* = 1042	
Insomnia ^a^	6.0 (3, 10)	7 (4, 10)	8.0 (5, 12)	<0.0001
Overweight and obese, N (%)	429 (25.4)	624 (37.0)	634 (37.6)	0.002
Stage at Diagnosis				
I, N (%)	295 (25.8)	448 (39.1)	402 (35.1)	0.31
II, N (%)	474 (28.5)	609 (36.6)	583 (35.0)	
III, N (%)	44 (29.5)	48 (32.2)	57 (38.2)	
ER+/PR+, N (%)	523 (28.5)	684 (37.2)	631 (34.3)	0.01
Post-Menopause, N (%)	654 (27.8)	881 (37.5)	817 (34.7)	0.82
Chemotherapy, N (%)	546 (26.5)	768 (37.2)	749 (36.3)	0.23
Radiation, N (%)	503 (27.7)	665 (36.6)	648 (35.6)	0.83
Smoking Status				
Never, N (%)	404 (25.4)	605 (38.0)	585 (36.7)	0.003
Past, N (%)	379 (30.8)	455 (36.9)	398 (32.3)	
Current, N (%)	30 (22.4)	45 (33.6)	59 (44.0)	
Alcohol Abstainer, N (%)	239 (25.7)	330 (35.5)	361 (38.8)	0.003
Education College or Greater, N (%)	484 (30.2)	624 (39.0)	491 (30.7)	<0.001

^a^ Continuous variables are presented as median (inter-quartile range). To test statistical significance among three groups of women with different pessimism scores, we used ANONVA for a continuous variable (variables labeled with ^a^, such as insomnia) and chi-squared test for a categorical variable (variables not labeled with ^a^, such as education). Abbreviations: WHEL study: the Women’s Healthy Eating and Living study, ER.: estrogen receptor-positive, PR: progesterone receptor-positive.

**Table 3:** Baseline characteristics according to total calorie intake in the WHEL study (*n* = 2960).

	Calorie Intake				
	Quartile 1	Quartile 2	Quartile 3	Quartile 4	*p*-Value
	<1363	1363< to <1603	1603< to <1876	≥1876	
	*n* = 550	*n* = 668	*n* = 777	*n* = 965	
Insomnia ^a^	7.0 (4.0, 11.0)	7.0 (4.0, 11.0)	7.0 (4.0, 11.0)	7.0 (4.0, 11.0)	0.94
Normal Weight, N (%)	231 (18.2)	315 (24.7)	355 (27.9)	372 (29.2)	0.002
Stage at Diagnosis					
I, N (%)	227 (19.8)	251 (21.9)	296 (25.9)	371 (32.4)	0.18
II, N (%)	301 (18.1)	390 (23.4)	429 (25.8)	546 (32.8)	
III, N (%)	22 (14.8)	27 (18.1)	52 (34.9)	48 (32.2)	
ER+/PR+, N (%)	354 (19.3)	408 (22.2)	479 (26.1)	597 (32.5)	0.48
Post-Menopause, N (%)	464 (19.7)	546 (23.2)	609 (25.9)	733 (31.2)	0.005
Chemotherapy, N (%)	369 (17.9)	455 (22.1)	555 (26.9)	684 (33.2)	0.43
Radiation, N (%)	333 (18.3)	395 (21.8)	498 (27.4)	590 (32.5)	0.10
Smoking Status					
Never, N (%)	303 (19.0)	359 (22.5)	432 (27.1)	500 (31.4)	0.37
Past, N (%)	217 (17.6)	286 (23.2)	309 (25.1)	420 (34.1)	
Current, N (%)	30 (22.4)	23 (17.2)	36 (26.9)	45 (33.6)	
Alcohol Abstainer, N (%)	259 (27.9)	228 (24.5)	216 (23.2)	227 (24.4)	<0.0001
Education College or Greater, N (%)	272 (17.0)	369 (23.1)	439 (27.5)	519 (32.5)	0.07

^a^ Continuous variables are presented as median (inter-quartile range). To test statistical significance among three groups of women with different total calorie intakes, we used ANONVA for a continuous variable (variables labeled with ^a^, such as insomnia) and chi-squared test for a categorical variable (variables not labeled with ^a^, such as education). Abbreviations: WHEL study: the Women’s Healthy Eating and Living study, ER: estrogen receptor-positive, PR: progesterone receptor-positive.

**Table 4:** Baseline characteristics according to PRAL score in the WHEL study (*n* = 2960).

	PRAL Score				
	Quartile 1	Quartile 2	Quartile 3	Quartile 4	*p*-Value
	<−19.49	−19.49< to <−6.94	−6.94< to <3.22	≥3.22	
	*n* = 434	*n* = 782	*n* = 881	*n* = 863	
Insomnia ^a^	6 (3, 10)	7 (4, 11)	7 (4, 11)	7 (4, 11)	0.002
Normal Weight, N (%)	259 (20.4)	393 (30.9)	338 (26.6)	283 (22.2)	<0.0001
Stage at Diagnosis					
I, N (%)	171 (14.9)	298 (26.0)	335 (29.3)	341 (29.8)	0.58
II, N (%)	241 (14.5)	450 (27.0)	506 (30.4)	469 (28.2)	
III, N (%)	22 (14.8)	34 (22.8)	40 (26.9)	53 (35.6)	
ER+/PR+, N (%)	271 (14.7)	492 (26.8)	566 (30.8)	509 (27.7)	0.0001
Post-Menopause, N (%)	369 (15.7)	644 (27.4)	700 (29.8)	639 (27.2)	0.0001
Chemotherapy, N (%)	287 (13.9)	515 (25.0)	631 (30.6)	630 (30.5)	0.02
Radiation, N (%)	267 (14.7)	494 (27.2)	521 (28.7)	534 (29.4)	0.46
Smoking Status					
Never, N (%)	230 (14.4)	419 (26.3)	476 (29.9)	469 (29.4)	0.27
Past, N (%)	194 (15.8)	326 (26.5)	364 (29.6)	348 (28.3)	
Current, N (%)	10 (7.5)	37 (27.6)	41 (30.6)	46 (34.3)	
Alcohol Abstainer, N (%)	143 (15.4)	234 (25.2)	282 (30.3)	271 (29.1)	0.70
Education College or Greater, N (%)	283 (17.7)	452 (28.3)	470 (29.4)	394 (24.6)	<0.0001

^a^ Continuous variables are presented as median (inter-quartile range). To test statistical significance among three groups of women with different PRAL scores, we used ANONVA for a continuous variable (variables labeled with ^a^, such as insomnia) and chi-squared test for a categorical variable (variables not labeled with ^a^, such as education). Abbreviations: WHEL study: the Women’s Healthy Eating and Living study, ER: estrogen receptor-positive, PR: progesterone receptor-positive, PRAL potential renal acid load.

**Table 5:** Pessimism, total calorie intake, and dietary acid load in relation to insomnia.

			Insomnia Score (≥9 vs. <9)	
			OR (95% CI)	OR (95% CI)
		Range	Age-Adjusted Model	Multivariable Model
**Pessimism**				
	Tertile 1	<5	Ref	Ref
	Tertile 2	5 to <6	1.17 (1.04–1.32)	1.14 (1.01–1.28)
	Tertile 3	≥6	1.64 (1.43–1.86)	1.57 (1.37–1.79)
	***p* for trend**		<0.001	<0.001
**Calorie Intake**		Range		
	Quartile 1	<1363	Ref	Ref
	Quartile 2	1363 to <1603	1.11 (0.99–1.26)	1.12 (0.98–1.27)
	Quartile 3	1603 to <1876	1.17 (1.03–1.34)	1.19 (1.04–1.36)
	Quartile 4	≥1876	1.14 (1.00–1.30)	1.17 (1.02–1.35)
	***p* for trend**		0.01	0.02
**PRAL (mEq/day)**		Range		
	Quartile 1	<−19.50	Ref	Ref
	Quartile 2	−19.50 to <−6.94	1.19 (1.05–1.34)	1.16 (1.02–1.32)
	Quartile 3	−6.94 to <3.22	1.29 (1.13–1.46)	1.24 (1.09–1.42)
	Quartile 4	≥3.22	1.31 (1.15–1.50)	1.21 (1.05–1.39)
	***p* for trend**		<0.001	0.02
**NEAP (mEq/day)**		Range		
	Quartile 1	<28.44	Ref	Ref
	Quartile 2	28.44 to <37.25	1.15 (1.02–1.30)	1.13(1.00–1.29)
	Quartile 3	37.25 to <46.90	1.27 (1.11–1.44)	1.22 (1.07–1.39)
	Quartile 4	≥46.90	1.24 (1.08–1.42)	1.14 (1.00–1.32)
	***p* for trend**		0.004	0.16

Covariates in the multivariable-adjusted model included caffeine intake, BMI, age, smoking status, estrogen receptor and progesterone receptor, co-morbidities, menopausal status, and education. Abbreviations: PRAL—potential renal acid load, NEAP—net endogenous acid production.
